# Dual-band Circular Polarizer Based on Simultaneous Anisotropy and Chirality in Planar Metamaterial

**DOI:** 10.1038/s41598-017-17976-w

**Published:** 2018-01-29

**Authors:** Yizhe Zhao, Anyong Qing, Yang Meng, Zelin Song, Chuan Lin

**Affiliations:** 1 School of Physical Electronics, University of Electronic Science and Technology of China, Chengdu, 610054 China; 2 The Institute of Optics and Electronics, The Chinese Academy of Sciences, Chengdu, 610209 China

## Abstract

Metamaterial of dual-square array is proposed to design a dual-band circular polarizer. The novel design of asymmetric unit cell and layout of duplicate arrays significantly enhances the coupling between electric and magnetic fields. Simulation and measurement results show that the polarizer presents wide angle circular dichroism and circular birefringence. Moreover, the polarization conversion of the proposed metamaterial changes with frequency, incident angle, and polarization of incident waves. The fundamental mechanism behind is concluded to be the angle-dependent chirality and dispersion of our novel design.

## Introduction

In recent years, metamaterials have been studied intensively for their special electromagnetic properties which are inexistent in natural materials. Application-oriented metamaterials’ effective permittivity and permeability^1,2^ can be designed for some amazing functions such as invisible cloaking^3^, diffraction-limit breaking imaging^4,5^ and perfect lens^6^.

Recently, metamaterials have been used to manipulate polarization of electromagnetic waves^7–11^, for example, circular dichroism (CD) and circular birefringence (CB). One of the critical points behind polarization manipulation by using metamaterials is thought to be the artificial chirality. Chirality in metamaterials results in cross-coupling between electric and magnetic fields.

Prominent chiral metamaterials with circularly polarized eigenmodes^12–25^ include twisted U-shape split ring resonators and twisted complementary split ring resonators. However, the working bandwidth of the polarization rotator based on these metamaterials is relatively narrow. To broaden bandwidth, an infrared broadband circular polarizer with periodic gold helix structure has been proposed^12^. It is later scaled down to microwave^13^ and terahertz^14^ region. Unfortunately, the thickness of the polarizers studied in^12–14^ is of the order of the resonant wavelength so that it is hard to integrate them. Some approaches^15–18^ to miniaturize the structure to subwavelength thickness have been proposed.

In this paper, metamaterial of dual-square array is proposed to design a dual-band circular polarizer. Compared with previous designs^25–27^, the proposed structure is simpler. It transforms incident linearly polarized wave into left/right-handed circularly polarized wave at two resonant frequencies. The differences of transformation between left-handed circular polarization (LCP) and right-handed circular polarization (RCP) waves are more than 15 dB at both resonant frequencies.

## Design Principle

**Figure 1:**
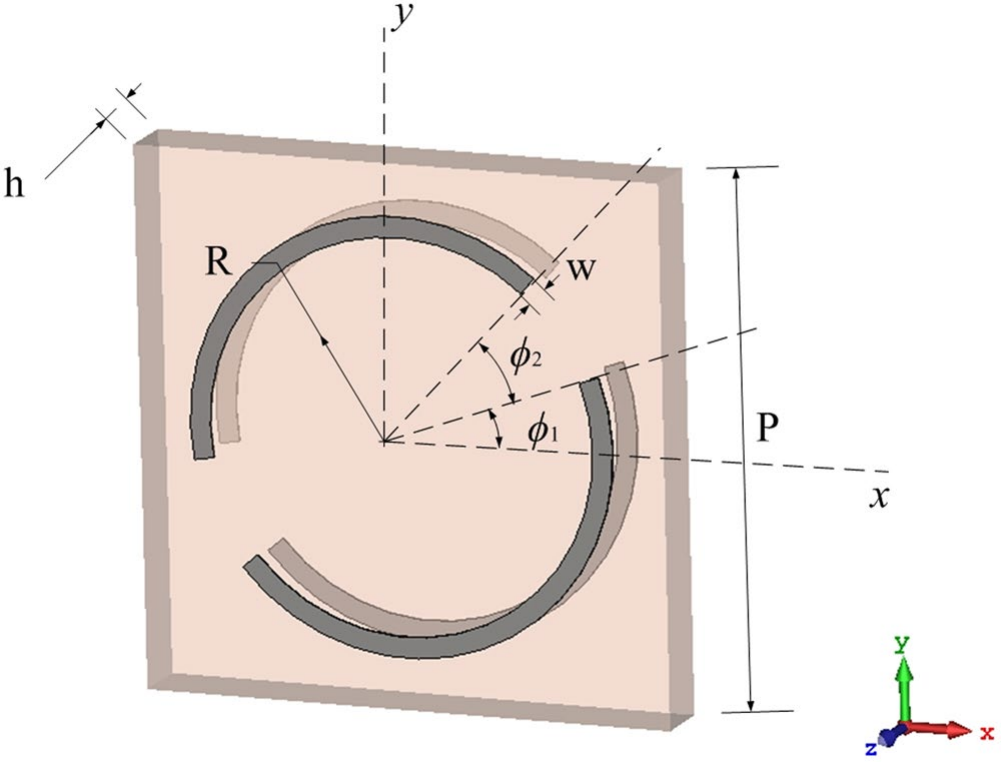
Two Aligned Unit Cells of the Dual-band Circular Polarizer.

**Figure 2:**
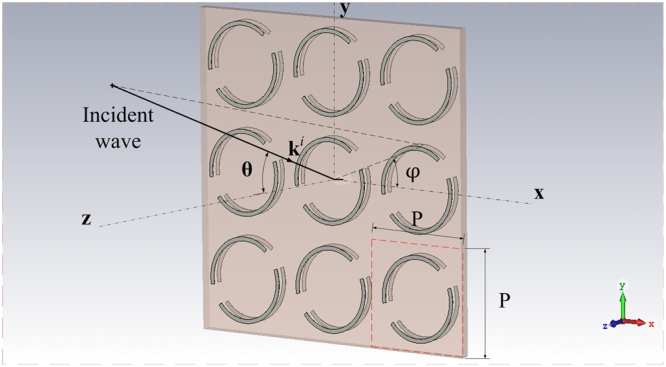
Oblique Incidence on the Metamaterial Array.

Inspired by the original multiband chiral metamaterial of twisted arc structure^24^, a dual-band circular polarizer with two aligned identical square arrays of periodicity *p* separated by a dielectric slab of thickness *h* is proposed in this paper. Two aligned unit cells of the arrays are shown in Fig. 1 while the configuration of arrays is shown in Fig. 2. In each unit cell, a centered PEC circle of radius *R* and width *w* is broken into two arcs at angle ϕ_1_ with an angle gap ϕ_2_.

The novel metamaterial highly asymmetric that results in is strong chirality. In addition, it is well known that helical- or spiral-like structure in most prominent metamaterial-based polarizers leads to the coupling of magnetic and electric field which is one of the fundamental mechanisms behind the circular dichroism and circular birefringence. To enhance the coupling, two aligned identical square arrays have been used. Consequently, circular dichroism and circular birefringence is achieved, as confirmed by numerical simulation and experiments.

The commercial software CST microwave studio is used to simulate the linear polarization transmission matrix **τ** of the metamaterial.

1 $$[\begin{array}{c}{E}_{\theta }^{t}\\ {E}_{\phi }^{t}\end{array}]=[\begin{array}{cc}{\tau }_{\theta \theta } & {\tau }_{\theta \phi }\\ {\tau }_{\phi \theta } & {\tau }_{\phi \phi }\end{array}]\,[\begin{array}{c}{E}_{\theta }^{i}\\ {E}_{\phi }^{i}\end{array}]$$

where ${{\bf{E}}}^{i}({\bf{r}})=({E}_{\theta }^{i}\hat{\theta }+{E}_{\phi }^{i}\hat{\phi }){e}^{j{{\bf{k}}}^{i}{\bf{r}}}$ is the incident plane wave, ${{\bf{k}}}^{i}={k}_{0}{\hat{k}}^{i},\,{k}_{0}=\omega \sqrt{\mu \varepsilon }\,,\,{\hat{k}}^{i}=sin{\theta }^{i}cos{\phi }^{i}\hat{x}+sin{\theta }^{i}$ $sin{\phi }^{i}\hat{y}+cos{\theta }^{i}\hat{z}$. ${{\bf{E}}}^{t}({\bf{r}})=({E}_{\theta }^{t}\hat{\theta }+{E}_{\phi }^{t}\hat{\phi }){e}^{-j{{\bf{k}}}^{t}{\bf{r}}}$ is the transmitted wave through the metamaterial, ${{\bf{k}}}^{t}={k}_{0}{\hat{k}}^{t},{\hat{k}}^{t}=$ $sin{\theta }^{t}cos{\phi }^{t}\hat{x}+sin{\theta }^{t}sin{\phi }^{t}\hat{y}+cos{\theta }^{t}\hat{z}$. In our simulation, ${\theta }^{i}={\theta }^{t},{\phi }^{i}={\phi }^{t}$.

The corresponding circularly polarized transmitted wave is

2 $$[\begin{array}{c}{E}_{+}^{t}\\ {E}_{-}^{t}\end{array}]=[\begin{array}{cc}1 & i\\ 1 & -i\end{array}][\begin{array}{c}{E}_{\theta }^{t}\\ {E}_{\phi }^{t}\end{array}]=[\begin{array}{cc}{\tau }_{\theta \theta }+i{\tau }_{\phi \theta } & {\tau }_{\theta \phi }+i{\tau }_{\phi \phi }\\ {\tau }_{\theta \theta }-i{\tau }_{\phi \theta } & {\tau }_{\theta \phi }-i{\tau }_{\phi \phi }\end{array}][\begin{array}{c}{E}_{\theta }^{i}\\ {E}_{\phi }^{i}\end{array}]={\bf{T}}[\begin{array}{c}{E}_{\theta }^{i}\\ {E}_{\phi }^{i}\end{array}]$$

where the subscripts + and − denote right- and left-handed circularly polarized waves respectively.

## Measurements and Discussions

**Figure 3:**
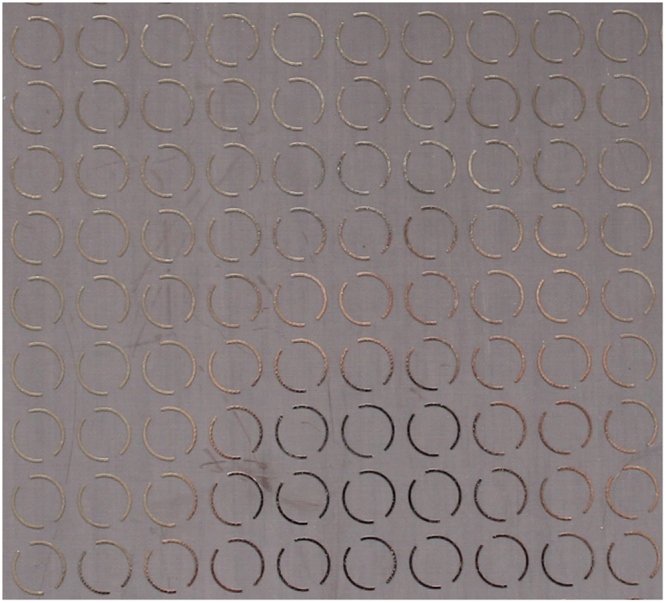
A Photo of the Fabricated Structure.

**Figure 4:**
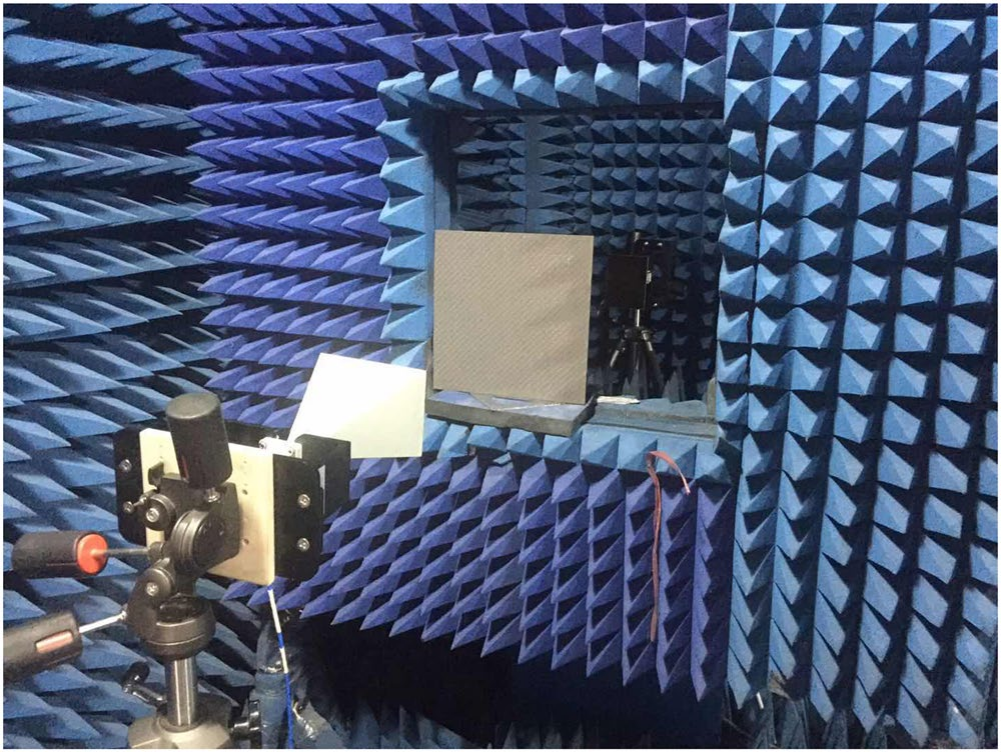
Experimental Setup.

The dual-band circular polarizer has been fabricated as shown in Fig. 3. The two 25 × 25 square arrays of periodicity *p* = 13 mm are separated by a Rogers 5880 (relative permittivity ${\varepsilon }_{r}=2.2$ and loss tangent tanδ = 0.0009) slab of thickness *h* = 1.524 mm. *R* = 5 mm, *w* = 0.5 mm, ϕ_1_ = 10°, ϕ_2_ = 40°. The experimental setup is shown in Fig. 4. The polarization transformation coefficient matrix **τ** of the fabricated polarizer has also been measured. The corresponding polarization transformation coefficient matrix **T** is subsequently obtained according to Eq. (2).

**Figure 5:**
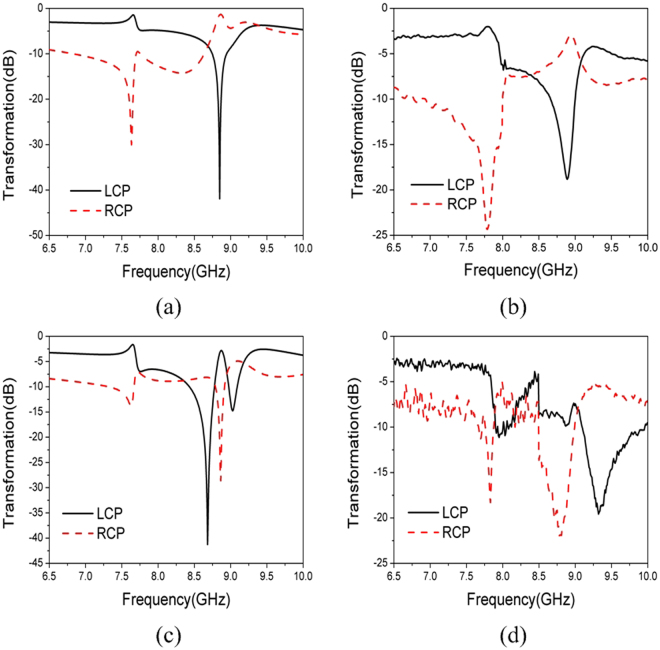
Transformation Coefficients of the Polarizer Illuminated by $\hat{y}(\hat{\phi })$-polarized Incident Wave. (**a**) simulation, *θ*^*i*^ = 50° and *φ*^*i*^ = 0° (**b**) measurement, *θ*^*i*^ = 50° and *φ*^*i*^ = 0°. (**c**) Simulation, *θ*^*i*^ = 50° and *φ*^*i*^ = 180° (**d**) measurement, *θ*^*i*^ = 50° and *φ*^*i*^ = 180°.

The simulated and measured transformation coefficients of the polarizer illuminated by $\hat{y}(\hat{\phi })$-polarized incident wave of *θ*^*i*^ = 50° and *φ*^*i*^ = 0° is show in Fig. 5(a),(b). Simulation and measurement agree fairly well. Two resonant frequencies at 7.68 GHz and 8.82 GHz are observed in Fig. 5(a). The transmitted field at 7.68 GHz is LCP and RCP at 8.82 GHz which is evident from the large difference between ${E}_{-}^{t}$ and ${E}_{+}^{t}$. LCP wave is observed at 7.76 GHz and RCP wave is seen at 8.9 GHz in Fig. 5(b). Both resonant frequencies are slightly shifted lower by about 0.1 GHz.

The simulated and measured transformation coefficients of the polarizer illuminated by $\hat{y}(\hat{\phi })$-polarized incident wave of *θ*^*i*^ = 50° and *φ*^*i*^ = 180° is show in Fig. 5(c),(d). The same resonant frequencies as Fig. 5(a) are present in Fig. 5(c). Both of these two resonant frequencies are also slightly shift by about 0.1 GHz in Fig. 5(d). Unlike the case in Fig. 5(a),(b), at both resonant frequencies, the transmitted field in Fig. 5(c),(d) is all LCP. Although only circular dichroism can be confirmed at lower resonant frequency, both circular dichroism and circular birefringence are presented at higher frequency. This proves that our novel metamaterial is dispersive and chiral. Moreover the chirality is angle-dependent.

**Figure 6:**
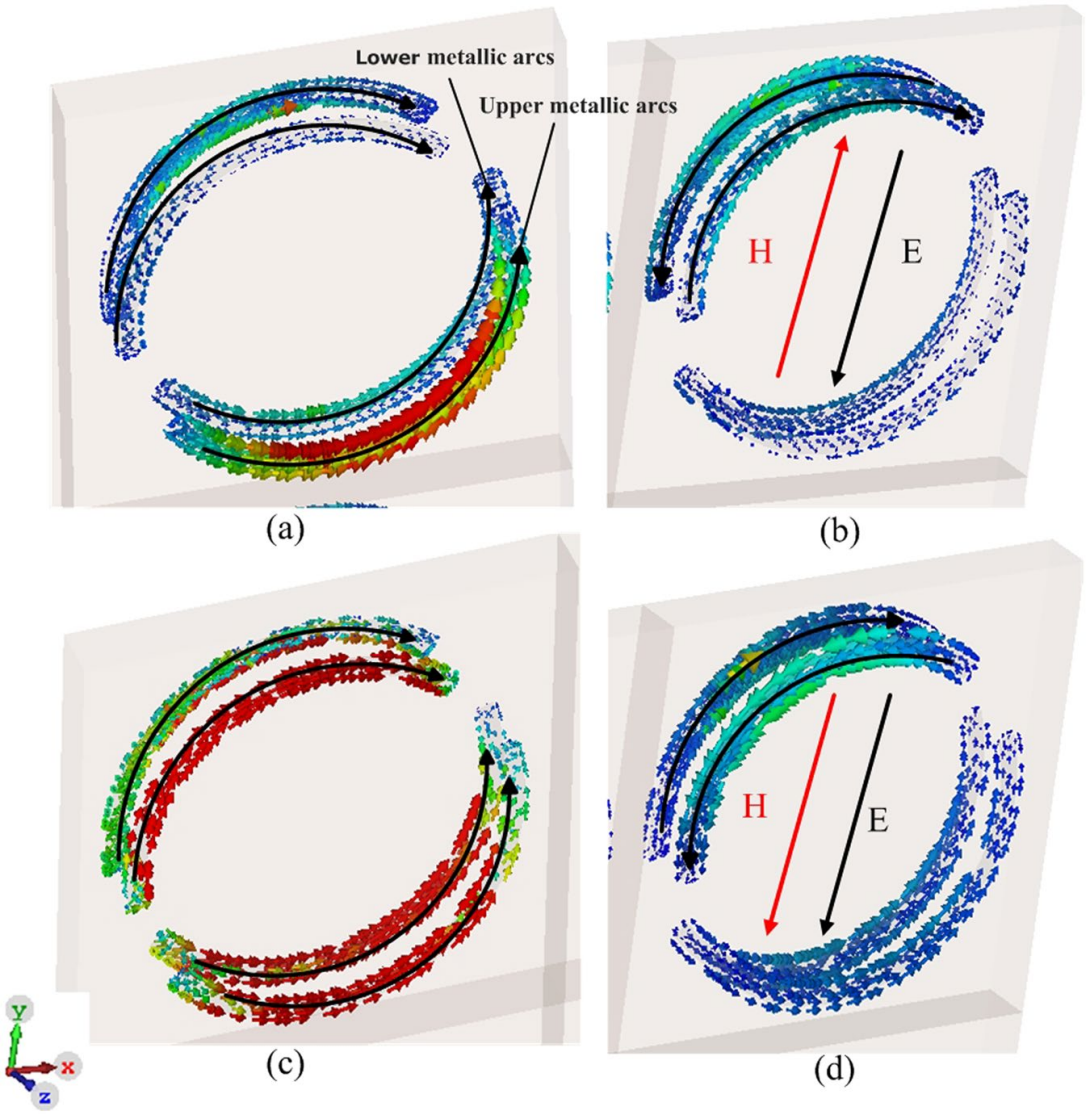
Surface Current Distributions of the Polarizer Illuminated by $\hat{y}(\hat{\phi })$-polarized Incident Wave. (**a**) 7.68 GHz, *θ*^*i*^ = 50° and *φ*^*i*^ = 0° and (**b**) 8.82 GHz, *θ*^*i*^ = 50° and *φ*^*i*^ = 0°. (**c**) 7.68 GHz, *θ*^*i*^ = 50° and *φ*^*i*^ = 180° (**d**) 8.82 GHz, *θ*^*i*^ = 50° and *φ*^*i*^ = 180°.

The surface current distributions at these two resonant frequencies are drawn in Fig. 6 to have a deeper understanding of the mechanism behind the observed circular dichroism and circular birefringence. As shown in Fig. 6(a),(c), at 7.68 GHz regardless of the incident azimuthal angle *φ*^*i*^, currents in the two arcs in each unit cell flow opposite to each other, while currents in the aligned arcs in the top and bottom arrays flow in the same direction. Therefore, there is no current loop in any unit cells. There is no current loop between the top and bottom arrays either. On the contrary as shown in Fig. 6(b),(d) at 8.82 GHz, currents in the two arcs in each and every unit cell form a loop. Current loops are also formed between the top and bottom arrays. In addition, the rotation of the current loops at *φ*^*i*^ = 0° are opposite to the corresponding current loops at *φ*^*i*^ = 180°.

Obviously, electric field couples differently with magnetic field in different frequencies and incident angles. For example, in Fig. 6(b), the induced magnetic field has a y-component that opposes the incident electric field, producing cross coupling between electric and magnetic fields in the metamaterial accordingly. However, in Fig. 6(d), the induced magnetic field has a y-component that coincides with the incident electric field. The resultant values of the chiral parameter *k* in Fig. 6(b),(d) are opposite to each other. This clearly reveals the physical origin of the circular dichroism and circular birefringence by our metamaterial.

**Figure 7:**
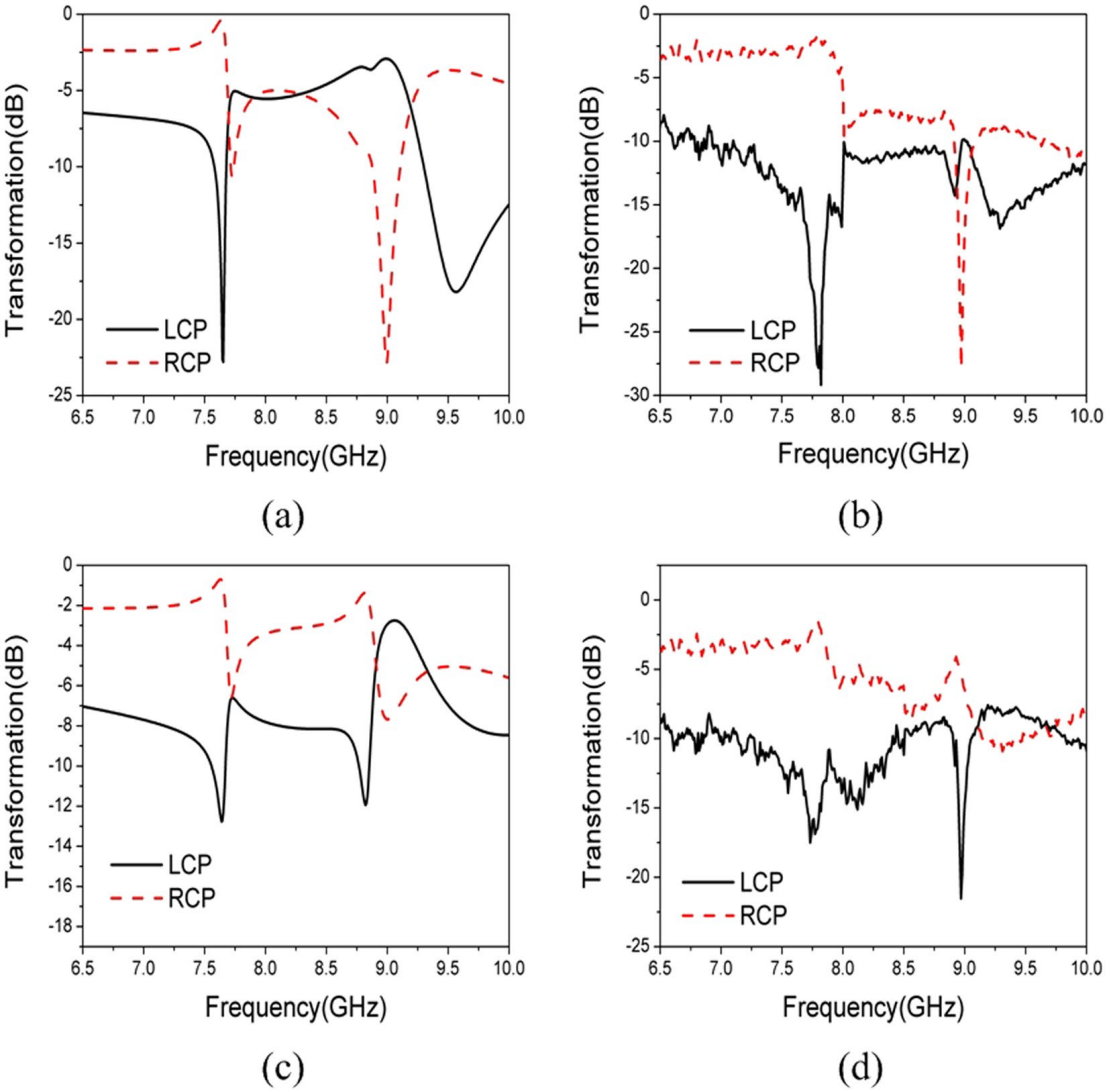
Transformation Coefficients of the Polarizer Illuminated by $\hat{\theta }$-polarized Incident Wave. (**a**) Simulation, *θ*^*i*^ = 50° and *φ*^*i*^ = 0° (**b**) measurement, *θ*^*i*^ = 50° and *φ*^*i*^ = 0°. (**c**) Simulation, *θ*^*i*^ = 50° and *φ*^*i*^ = 180° (**d**) measurement, *θ*^*i*^ = 50° and *φ*^*i*^ = 180°.

The simulated and measured transformation coefficients of the polarizer illuminated by $\hat{\theta }$-polarized incident wave of *θ*^*i*^ = 50° and *φ*^*i*^ = 0° is show in Fig. 7(a),(b). Likewise the simulation and measurement agree fairly well. Three resonant frequencies at 7.63 GHz, 9.00 GHz, and 9.58 GHz are observed in Fig. 7(a). The transformed field at 9.0 GHz is LCP, while are RCP at 7.63 GHz and 9.58 GHz. It can be seen that transmitted wave is RCP at 7.80 GHz and 9.29 GHz, and LCP at 9.00 GHz in Fig. 7(b). All of these three resonant frequencies are shifted lower by about 0.3 GHz.

The simulated and measured transformation coefficients of the polarizer illuminated by $\hat{\theta }$-polarized incident wave of *θ*^*i*^ = 50° and *φ*^*i*^ = 180° is show in Fig. 7(c),(d). The structure also resonates at three frequencies, 7.63 GHz, 8.85 GHz, and 9.12 GHz by simulation, while 7.75 GHz, 8.96 GHz and 9.24 GHz by measurement. The difference between $|{T}_{-\theta }|$ and $|{T}_{+\theta }|$ at the highest resonant frequency is relatively smaller that no further discussion is made at this frequency.

By comparing Fig. 7(a),(b) with Fig. 7(c),(d), once again only circular dichroism can be confirmed at lower resonant frequency while both circular dichroism and circular birefringence are presented at higher frequency. This feature proves that our novel metamaterial is dispersive and angle-dependent chiral.

**Figure 8:**
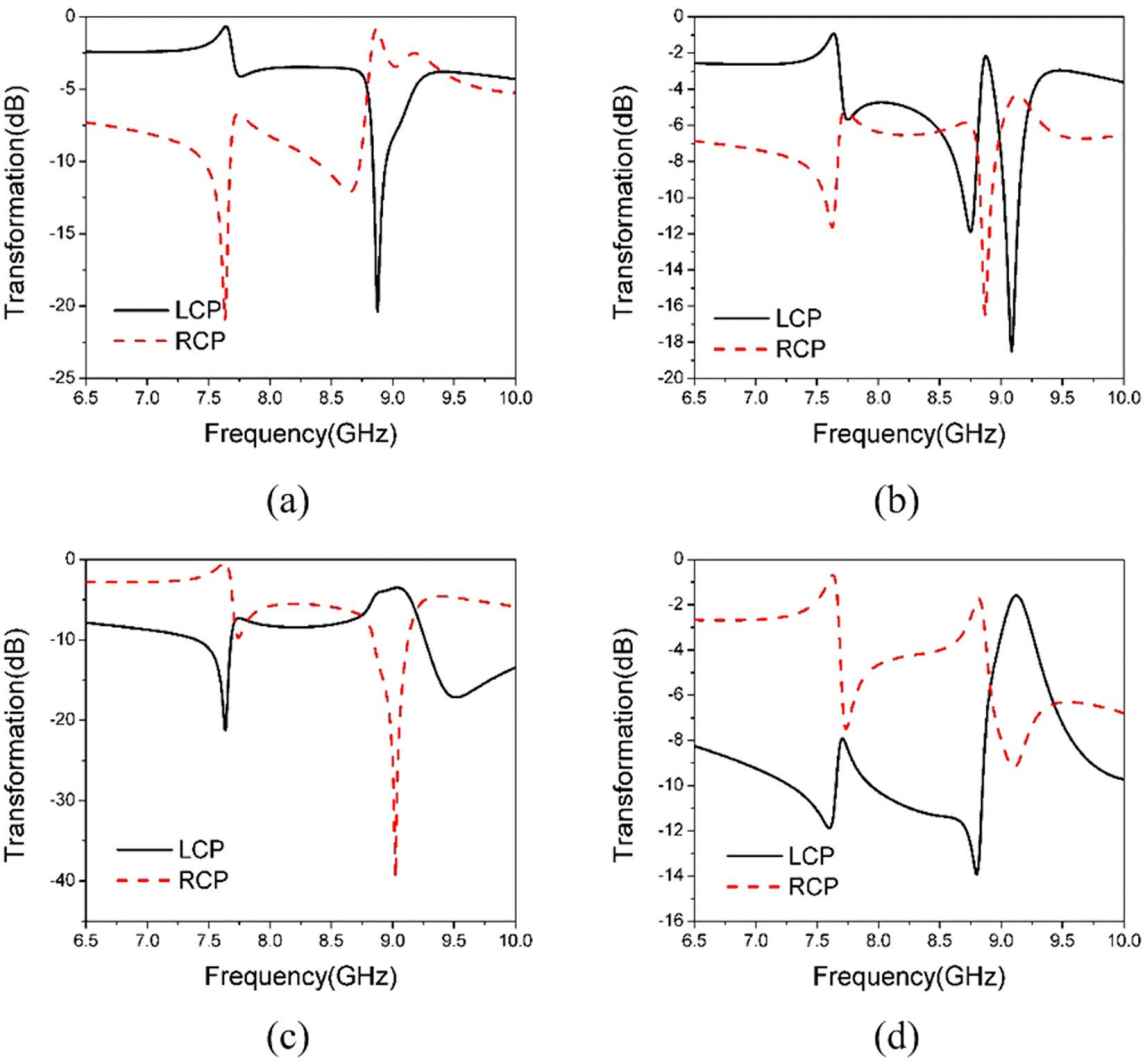
Simulated Transformation Coefficients of the Polarizer. (**a**) $\hat{y}(\hat{\phi })$-polarized incident wave, *θ*^*i*^ = 30° and *φ*^*i*^ = 0° (**b**) $\hat{y}(\hat{\phi })$-polarized incident wave, *θ*^*i*^ = 30° and *φ*^*i*^ = 180°(**c**) $\hat{\theta }$-polarized incident wave, *θ*^*i*^ = 30° and *φ*^*i*^ = 0° (**d**) $\hat{\theta }$-polarized incident wave, *θ*^*i*^ = 30° and *φ*^*i*^ = 180°.

More simulations have been performed to study the dependence of circular dichroism and circular birefringence of our metamaterial on the incident angle. Remarkable polarization transformation has been observed for elevation angles *θ*^*i*^ larger than 30°. Beyond that, the difference between the LCP and RCP components in the transmitted wave becomes smaller and smaller as shown in Fig. 8.

## Conclusion

A dual-band circular polarizer based on a novel metamaterial has been presented in this paper. The metamaterial implements a novel asymmetric unit cell and layout of duplicate arrays. Wide angle circular dichroism and circular birefringence have been observed in both numerical simulation and measurement. The fundamental mechanism behind is concluded to be the angle-dependent chirality and dispersion of our novel metamaterial. The multi-band metamaterial could be applied in microwave field and might be promising in terahertz and optical region.

## References

[1] Pendry JB (1996). “Extremely low frequency plasmons in metallic mesostructures. Phys. Rev. Lett..

[2] Smith DR, Pendry JB, Wiltshire MCK (2004). “Metamaterials and negative refractive index. Science.

[3] Zhu JF (2015). “Three-dimensional magnetic cloak working from d.c. to 250 kHz. Nat. Commun..

[4] Wang P (2013). “Far-field imaging of non-fluorescent species with subdiffraction resolution. Nat. Photon..

[5] Luo XG, Ishihara T (2004). “Surface plasmon resonant interference nanolithography technique. Appl. Phys. Lett..

[6] Tomáš Tyc, Zhang X (2011). “Forum Optics: Perfect lenses in focus. Nature.

[7] Cui JH (2016). “Dynamical manipulation of electromagnetic polarization using anisotropic meta-mirror. Sci. Rep..

[8] Ma, X. L. *et al*. An Active Metamaterial for Polarization Manipulating. *Adv. Optical Mater*. **2**(10), 945–949 (2014).

[9] Guo YH (2015). “Dispersion management of anisotropic metamirror for super-octave bandwidth polarization conversion. Sci. Rep..

[10] Ma XL (2012). “Multi-band circular polarizer using planar spiral metamaterial structure. Opt. Express.

[11] Pu MB (2013). “Anisotropic meta-mirror for achromatic electromagnetic polarization manipulation. Appl. Phys. Lett..

[12] Gansel JK (2009). “Gold helix photonic metamaterial as broadband circular polarizer. Science.

[13] Wu C (2011). “Metallic helix array as a broadband wave plate. Phys. Rev. Lett..

[14] Yu Y (2011). “Broadband optical circular polarizers in the terahertz region using helical metamaterials. J. Opt..

[15] Li ZF (2010). “Chiral metamaterials with negative refractive index based on four “U” split ring resonators. Appl. Phys. Lett..

[16] Zhao, R. *et al*. Conjugated gammadion chiral metamaterial with uniaxial optical activity and negative refractive index. *Phys. Rev. B*, **83**, 3 (2011).

[17] Xu HX (2013). “Compact dual-band circular polarizer using twisted Hilbert-shaped chiral metamaterial. Opt. Express..

[18] L. Xie HL, Yang X, Huang ZL (2013). “Multi-band circular polarizer using archimedean spiral structure chiral metamaterial with zero and negative refractive index. Prog. Electromagn. Res..

[19] Yu S (2016). “Low-dimensional optical chirality in complex potentials. Optica.

[20] Yu S, Piao X, Park N (2016). “Acceleration toward polarization singularity inspired by relativistic E × B drift. Sci. Rep..

[21] Ji R (2016). “Broadband circular polarizers constructed using helix-like chiral metamaterials. Nanoscale.

[22] Abadi SMAMH, Behdad N (2016). “Wideband linear-to-circular polarization converters based on miniaturized-element frequency selective surfaces. IEEE Trans. on Ant. and Propag..

[23] Piao X (2015). “Spectral separation of optical spin based on antisymmetric Fano resonances. Sci. Rep..

[24] Singh R (2010). “Highly tunable optical activity in planar achiral terahertz metamaterials”. Opt. Express.

[25] Plum E, Fedotov VA, Zheludev NI (2009). “Metamaterials: Optical Activity Without Chirality. Phys. Rev. Lett..

[26] Decker M (2010). “Twisted split-ring-resonator photonic metamaterial with huge optical activity. Opt. Lett..

[27] WU JF (2013). “Free-standing terahertz chiral meta-foils exhibiting strong optical activity and negative refractive index. Appl. Phys. Lett..

